# Peripheral Nerve Sheath Tumor: A Diagnostic and Therapeutic Challenge

**DOI:** 10.7759/cureus.56601

**Published:** 2024-03-20

**Authors:** Deviprasad Sulli, Chandni Shankar, Shruti G Raikar

**Affiliations:** 1 Plastic Surgery, Yenepoya Medical College, Mangalore, IND; 2 Plastic and Reconstructive Surgery, Yenepoya Medical College, Mangalore, IND

**Keywords:** neurilemmoma, neurofibroma, schwannoma, hoffman tinel sign, peripheral nerve sheath tumors

## Abstract

Introduction: Peripheral nerve tumors are a group of rare soft tissue tumors of neuro-ectodermal origin. Although the majority of them are benign in nature, up to 10% can be malignant. The symptoms depend on the site, size, and structures compressed by the tumor.

Aim: To highlight the heterogeneity of signs and symptoms and their presentations, which has often made it difficult for the attending physician to accurately diagnose and direct the patient toward appropriate treatment.

Methods: Eight patients treated at our tertiary care hospital between 2015 and 2022 were included in this study. They were evaluated in detail. Treatment was surgical. The patients underwent complete excision of the tumor under magnification to help preserve the adjacent neurovascular bundle. All patients were followed up post-operatively to document the status of their symptoms.

Results: The average duration prior to referral to our hospital was 13 months. Seven subjects had pain at presentation, one had neurological deficit. Seven also complained of swelling. Five of the eight lesions were schwannoma, two neurofibroma and one showed malignant histology. Post-operatively, Hoffman Tinel signs improved in all six subjects. five of the seven subjects were completely pain-free, and the other two had a reduction in symptoms.

Conclusions: Early diagnosis and referral to a specialist center are needed to achieve satisfactory outcomes while treating peripheral nerve tumors. Proliferative lesions should be treated surgically in specialist centers by experienced doctors with appropriate skills and equipment for microsurgical procedures to ensure full recovery.

## Introduction

Peripheral nerve tumors are a group of rare soft tissue tumors that are of neuro-ectodermal origin [1-3]. Although the majority of them are benign in nature, up to 10% can show malignant histology [4-6]. These tumors can develop along any of the peripheral nerves. The symptoms, depending on the site, size, and structures compressed by the tumor, can vary widely. Methods used to diagnose peripheral nervous system tumors include plain radiographs, ultrasound examination, computed tomography, magnetic resonance imaging, and positron emission tomography [7-10]. Treatment of peripheral nerve sheath tumors is predominantly surgical [11-13]. The reason behind our work is to highlight the heterogeneity of signs and symptoms and their presentations, which has often made it difficult for the attending physician to accurately diagnose and direct the patient toward appropriate treatment.

## Materials and methods

The study was conducted in the tertiary care hospital at Yenepoya Medical College, Mangalore, India. All patients with peripheral nerve sheath tumors during the period between 2015 and 2022 were included in the study. A detailed history was recorded emphasizing the type and duration of symptoms, the first appearance of swelling, and the rate of increase in size. History of previous conservative/ surgical interventions or any trauma was also documented. A thorough clinical examination was performed to look for characteristics of the swelling, the presence or absence of the Hoffman Tinel sign, and any sensory or motor deficits in the affected limb/site. All these details were documented in a pre-designed proforma.

Imaging study in the form of magnetic resonance imaging was performed in all the cases to demonstrate the tumor, its precise size and location as well as its relation to the peripheral nerve and the other adjacent structures in the region. Treatment in all cases was surgical. The surgeries were performed by an experienced plastic surgeon under general, regional, or local anesthesia, depending on the site of the tumor. The patients underwent complete excision of the tumor. The surgeries were performed under loupe magnification (3.5X) in order to help preserve the adjacent neurovascular bundle and prevent any permanent neurological deficits. Intra-operatively, a nerve stimulator was used as a guide during neurolysis to preserve nerve fibers in close proximity to the tumor.

All patients were re-examined, post-operatively, prior to discharge and at two weeks and three months post-operatively. During writing this review, the status of their presenting symptoms was assessed. A neurological examination was done to look for sensory/motor deficits, if any, in the area supplied by the nerve in question.

## Results

The study cohort included eight subjects consisting of four males and four females aged between 17 and 70 years (average age of 40 years) (Table 1). Three subjects had tumors located on the upper limb. Three had lower limb peripheral nerve involvement. One was from the carotid sheath with another arising from the upper lip.

**Table 1:** Demographics and clinical features m: Male; f: Female

Case	Sex	Age	Swelling location	Swelling duration (months)	Symptoms	Duration of symptoms (months)	Duration since first physician consultation (months)	Tumor location	Tumor size (cm)
1	m	32	Left ring finger	noticed on clinical examination	Pain, paresthesia	6	4	Left ring finger near the radial neurovascular bundle	1x0.5
2	f	46	The right side of the neck	6	Pain along the limb	24	18	C5, C6 nerve root level	3x3
3	m	23	The anteromedial aspect of the right arm	8	Weakness of the right hand	12	10	Distal forearm median nerve	1x1.5
4	f	65	Posterior thigh	24	Left lower limb pain	48	45	Posterior aspect of sciatic nerve	10x6
5	f	17	The anteromedial aspect of the thigh	12	Pain in the anterior aspect of the knee	12	12	Subcutaneous swelling anteromedial thigh	1x1
6	m	25	Upper lip	6	Nil	-	5	Vermilion of the upper lip, just to the left of the midline	2x2.5
7	m	70	Buttock	5	Pain along the lower limb	12	10	Sciatic nerve sheath	4x5
8	f	42	Neck	7	-	16	6	Carotid nerve sheath	3x3

All except one subject had pain as the presenting complaint. One subject presented with complaints of weakness in the area of distribution of the involved nerve. The duration of symptoms ranged from six to 48 months (18 months average). The subjects themselves complained of swelling in seven of the eight instances with the swelling being present for an average of 10 months (5-24 months range) (Table 1).

All these subjects had previously consulted a primary care physician/ orthopedic specialist for their complaints and were on regular/intermittent treatment by their doctor for an average duration of 13 months prior to referral to our tertiary care hospital. None of these patients underwent surgical treatment. They were managed with analgesics and/or physiotherapy.

**Figure 1:**
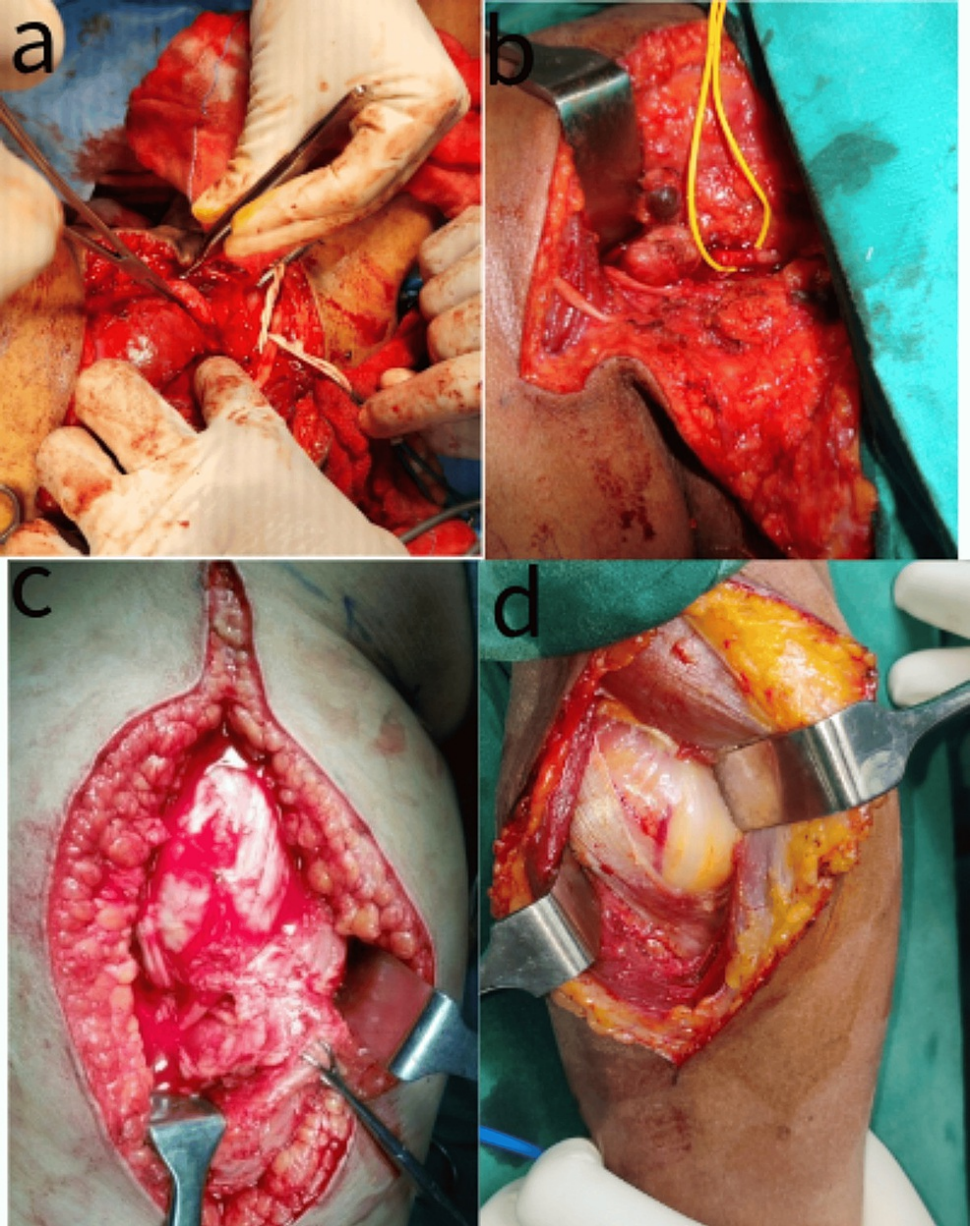
Intra-operative images (a) Brachial plexus nerve sheath tumor; (b) Neck schwannoma sitting between C5 and C6 nerve roots; (c) Sciatic nerve sheath tumor with tumor stretching out the tibial and peroneal components of the nerve; (d) Posterior interosseous nerve sheath tumor.

**Figure 2:**
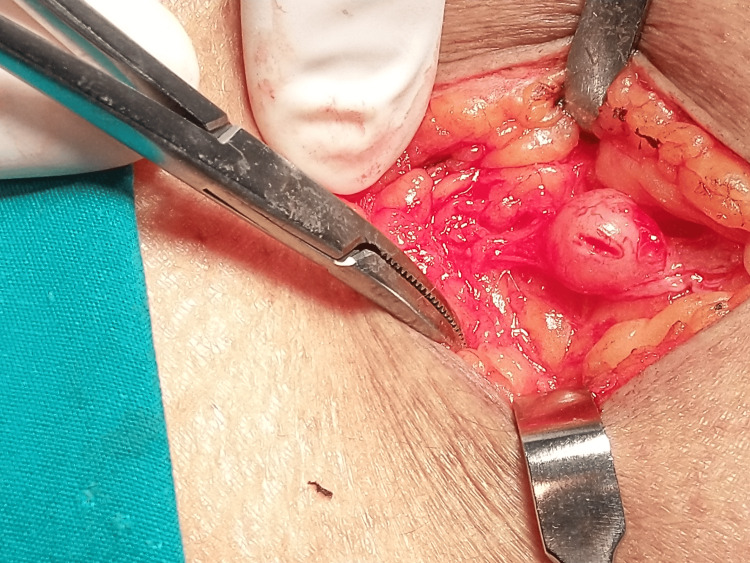
Intra-operative image (close view) The Image shows a cutaneous nerve sheath tumor that needed end-to-end neurorrhaphy after excision to restore nerve continuity.

Upon clinical examination, six subjects were found to have a positive Hoffman Tinel sign. All patients underwent MRI to define the tumor and aid in surgical planning. All subjects underwent surgical excision under magnification to ensure complete removal with preservation of the nerve trunk. In cases where the tumor was clearly seen separate from the nerve fibers or seen arising from the nerve sheath and not adherent to the nerve fibers, a nerve stimulator was not used. Four cases needed the aid of a nerve stimulator to confirm preservation of the involved nerve, one of them being a nerve sheath tumor involving the brachial plexus, as seen in Figures 1a, 1b. The sciatic nerve sheath tumor seen in Figure 1c and posterior interosseous nerve sheath tumor seen in Figure 1d needed intraneural dissection to ensure complete removal, and one case needed end-to-end neurorrhaphy to repair the nerve at the end of the excision (Figure 2).

Five of the eight lesions were schwannoma on histopathology, two revealed neurofibroma, and one showed malignant histology (Table 2).

**Table 2:** Histopathology and surgical outcome pre-op: Pre-operation

Case Number	Histopathology	Period of Follow-up	Recurrence	Symptoms	Tinel sign
1	Schwannoma	36	no	Nil	Resolved
2	Schwannoma	48	no	Nil	Resolved
3	Schwannoma	3	no	improving but lost to follow-up	Resolved
4	Malignant tumor	12	no	improved	Resolved
5	Schwannoma	16	no	Nil	Resolved
6	Neurofibroma	18	no	Nil	Absent pre-op
7	Neurofibroma	20	no	improved	Resolved
8	Schwannoma	15	no	nil	absent pre-op

Post-operatively, the first assessment showed clinical improvement in Hoffman Tinel signs in all six subjects who had a positive sign pre-operatively. Five of the seven subjects were completely pain-free, and the other two had a reduction in symptoms at two weeks post-operatively. The two subjects with incomplete resolution of symptoms showed further improvement at three months. The average follow-up period of these patients was 21 months with the shortest being three months (Table 2).

## Discussion

The prevalence was equal among men and women among our patients. The mean age at treatment of our patients was 42.5 female patients and 37.5 years for male patients. Though the literature suggests these tumors to be more common in the upper limbs, our patients had equal distribution in the upper and lower limbs [3].

The average duration of symptoms prior to seeking specialist treatment was 18 months (a range of six to 48 months). Most patients received treatment from the primary care physician at least once where the primary cause for the subjects’ symptoms was missed, resulting in a delay in seeking definitive care. The discovery of a swelling, however, decreased the delay to about 10 months. One of the causes for delay in treatment was because they were advised conservative management by the primary care physician because the tumor was clinically benign and for fear of neurological deficit following surgical excision. In our subjects, tumors derived from major peripheral nerves were more common (five subjects) when compared to small nerve branches. Small nerve branch-derived tumors were mainly predominantly located in the extremities of the limbs

According to published literature, up to 90% of peripheral nerve tumors are benign [14,15]. Malignant neoplasms originating from nerve sheaths formed anywhere between 9 to 15 % of the total number according to various studies [3,16,17]. In our series too, benign tumors were the predominant histopathology (seven out of eight tumors), constituting 87.5% of the total number of hyperplastic lesions, consistent with the available literature.

It was observed by Cashen et al. that female subjects had a greater predisposition for developing malignant tumors [18]. The only malignant neoplasm arising from nerve sheath (malignant peripheral nerve sheath tumors (MPNST)) diagnosed was a 65-year-old lady with a tumor arising from the posterior aspect of her thigh. However, our sample is too small to add any further strength to this view.

Apart from detected swelling, the most common clinical finding in the preoperative period was a positive Hoffman Tinel sign. It was observed in six out of the eight patients. Although the Hoffman Tinel sign can occur due to external compression of the tumor on the nerve trunk, it is more likely to occur when the tumor itself is arising from the nerve trunk [10,11,19,20]. Paresthesia occurred in four patients and sensory deficit in only one patient. Studies have shown a much higher incidence of paresthesia and sensory deficit among peripheral neural sheath tumors (PNSTs) [2,21]. No tumor recurrence after surgical treatment has been recorded over a mean follow-up period of 24 months in our case series.

Pain relief was observed in all patients, but 37.5% (three cases) had incomplete relief of symptoms, and the Hoffman Tinel sign was negative in six out of seven (85%) patients who presented these symptoms before the operation. High rates of resolution of paresthesia and motor deficits were also reported in the literature. Ozdemir et al., in their clinical material, observed improvement in clinical symptoms in all but one case [21]. Kang et al. study of 20 patients with schwannomas of the upper limb also noted improvement in all but one patient [22]. Knight et al. observed significant post-operative functions improving in 193 of the 198 patients [23]. In our series of cases, it was possible to remove the tumor without damage to the fascicular structure in all seven cases, with one case needing end-to-end neurorrhaphy, but none of the cases had any serious post-surgical worsening of symptoms or development of new neurological deficits.

Many factors, such as tumor location and size, histopathological type, duration and severity of symptoms, and choice of microsurgical technique to achieve complete removal with preservation of the nerve, affect the outcome of surgical treatment [4,12,14]. The diversity of the available clinical material makes it impossible to draw any direct comparisons between the outcomes. However, the results of our series of cases can be said to be comparable to those presented in similar studies [11,21,24-26].

## Conclusions

Early diagnosis and prompt referral for specialist care are paramount in swift and successful treatment of these rare tumors. Proliferative lesions of peripheral nerves should be treated surgically in specialist centers by experienced doctors with appropriate skills and equipment that enable the performance of microsurgical procedures. This is of great importance to achieve satisfactory outcomes while treating peripheral nerve tumors and reducing patient suffering. Malignant peripheral nerve tumors need to be treated with local radiotherapy after surgical excision, and these patients should be on regular follow-ups.
